# Prenylated p-Coumaric Acid Derivatives Mitigate Neurobehavioral and Neuroinflammatory Alterations Associated with Experimental Colitis

**DOI:** 10.3390/ijms27135929

**Published:** 2026-07-01

**Authors:** Camila A. Cazarin, Bruna Longo, Eduarda R. Bauer, Morgana S. Machado, Maria I. Basílio, Tauani C. S. França, Thiago F. de Q. e Silva, Benhur J. Cury, Larissa Venzon, Ana C. dos Santos, Heloísa I. Eisendecker, Luiza F. Corsi, Alex W. Valachinski, Sérgio F. de Andrade, Victor P. Ribeiro, Matheus H. Tanimoto, Jairo K. Bastos, Luísa M. da Silva, Márcia M. de Souza

**Affiliations:** 1 Pharmaceutical Sciences Postgraduate Program, University of Vale do Itajaí, Itajaí 88302901, SC, Brazilmsouza@univali.br (M.M.d.S.); 2 Laboratory of Pharmacology Applied to the Gastrointestinal Tract and its Interactions, Department of Pharmacology, Federal University of Santa Catarina, Florianópolis 88037000, SC, Brazil; 3 Research Institute for Medicines (iMed.ULisboa), Faculty of Pharmacy, Universidade de Lisboa, 1649-003 Lisbon, Portugal; 4 Department of Pharmacy, Pharmacology and Health Technologies, Faculty of Pharmacy, Universidade de Lisboa, Av. Prof. Gama Pinto, 1649-003 Lisbon, Portugal; 5 School of Pharmaceutical Sciences of Ribeirão Preto, University of São Paulo, Ribeirão Preto 14040903, SP, Brazil

**Keywords:** inflammatory bowel disease, neuroinflammation, Brazilian green propolis

## Abstract

Inflammatory bowel disease is an inflammatory disorder associated with systemic immune activation, contributing to neuroinflammation, behavioral impairments and disruption of the gut–brain axis. The present study investigated the effects of p-Coumaric acid derivatives: Artepillin C (ART-C), Baccharin (BAC), and Drupanin (DRU) on colonic damage, behavioral alterations, and oxidative stress in a dextran sulfate sodium (DSS)-induced colitis by administration of 3% DSS. Mice were treated with p-Coumaric acid derivatives (0.3, 1, or 3 mg/kg, p.o.), and disease activity index and colon length were evaluated as clinical parameters. Behavioral assessments included the open field test, novel object recognition test, elevated plus maze, and tail suspension test. Oxidative stress and inflammatory markers were quantified in colon, serum, cortex, and hippocampus, alongside histological analysis of colonic tissue. DSS administration induced clinical and histopathological alterations, increased oxidative stress, and impaired recognition memory, as well as anxiety- and depressive-like behaviors. p-Coumaric acid derivatives attenuated colonic damage, preserved tissue architecture, improved recognition memory, and reduced anxiety- and depressive-like behaviors, particularly at higher doses. These effects were associated with modulation of antioxidant defenses and reduction of lipid peroxidation and inflammatory markers. p-Coumaric acid derivatives exert protective effects in DSS-induced colitis, highlighting their potential as therapeutic agents for intestinal and neurobehavioral alterations associated with IBD.

## 1. Introduction

Inflammatory bowel disease (IBD) is a chronic, relapsing disorder of the gastrointestinal tract characterized by impaired barrier integrity, aberrant immune activation, and microbial dysbiosis [1]. Although classically viewed as a localized gut condition, IBD exerts systemic effects. Increased intestinal permeability facilitates the translocation of microbial products, such as lipopolysaccharides, into the circulation, where they amplify pro-inflammatory signaling and contribute to extra-intestinal complications [1,2]. These systemic alterations extend to the central nervous system (CNS), suggesting that IBD may act as a trigger for neurological dysfunction [3].

A central mechanism linking gut inflammation and the brain is the microbiota–gut–brain axis, a bidirectional communication system that integrates microbial, immune, endocrine, and neural signals [4]. Disruption of this axis due to dysbiosis and chronic intestinal inflammation enhances peripheral cytokine release, impairs blood–brain barrier function, and promotes microglial and astrocytic activation within the CNS [5]. However, it remains debated whether these processes primarily drive neurodegeneration or simply exacerbate ongoing pathology.

This controversy is particularly relevant to Alzheimer’s disease (AD). While the extracellular accumulation of amyloid-β plaques and intracellular tau tangles remain its classical hallmarks [6], mounting evidence implicates systemic inflammation and gut dysbiosis as significant modulators of disease onset and progression [7,8]. Some studies suggest that intestinal inflammation may act as an initiating event for AD-related pathology [9], while others argue it accelerates disease only in genetically or environmentally predisposed individuals [10]. These diverging hypotheses underscore the complexity of AD pathogenesis and highlight the urgent need for therapeutic approaches capable of modulating both peripheral and central inflammatory pathways.

Natural bioactive compounds with dual anti-inflammatory and neuroprotective potential are emerging as attractive candidates in this context. Brazilian green propolis, a resinous product rich in prenylated derivatives of p-Coumaric acid such as Artepillin C, Baccharin, and Drupanin, has demonstrated potent immunomodulatory, antioxidant, and neuroprotective properties [11,12,13]. By acting at the interface between gut-derived systemic inflammation and neuroinflammatory cascades, these compounds may offer a multitarget strategy to mitigate the pathological processes underlying both IBD and AD.

The present study investigates the effects of Artepillin C, Baccharin, and Drupanin in an experimental model of IBD-associated systemic and central inflammation. By simultaneously evaluating gastrointestinal, neuroinflammatory, and behavioral outcomes, we aim to clarify their potential as therapeutic agents bridging the gap between intestinal disorders and neurodegeneration.

## 2. Results

### 2.1. p-Coumaric Acid Derivatives Attenuate DSS-Induced Colitis Severity Evidenced in DAI Scores and Colon Length

**Figure 1:**
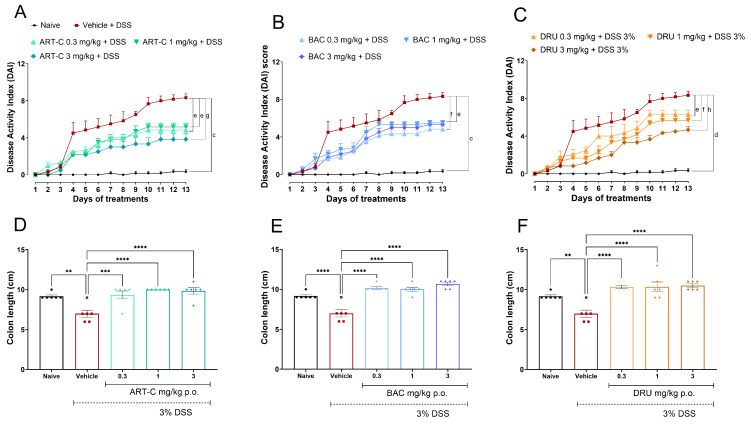
Effects of p-Coumaric acid derivatives on disease activity index (DAI) and colon length in DSS-induced colitic mice. The disease activity index (DAI) was monitored daily in mice treated with Vehicle (1% Tween 80 + water), (**A**) Artepillin C (ART-C), (**B**) Baccharin (BAC), or (**C**) Drupanin (DRU) at doses of 0.3, 1, and 3 mg/kg (p.o.). Dextran sulfate sodium (DSS, 3%) was administered for seven consecutive days to induce colitis, and treatments were administered concomitantly with DSS from the onset of colitis induction and maintained for 13 consecutive days. Colon length was measured at the end of the experimental protocol in mice treated with (**D**) ART-C, (**E**) BAC, and (**F**) DRU. Data are expressed as mean ± SEM (N = 6). Statistical analysis was performed using two-way ANOVA followed by Tukey’s post hoc test for DAI analysis and one-way ANOVA followed by Tukey’s post hoc test for colon length analysis. Letters indicate statistically significant differences. Letters c–d represent comparisons versus the Naive (non-colitic) group, and letters e–h represent comparisons versus the vehicle-treated colitic group: e (*p* < 0.05); f (*p* < 0.01); c, g (*p* < 0.001); d, h (*p* < 0.0001). Asterisks indicate statistical significance (** *p* < 0.01; *** *p* < 0.001; **** *p* < 0.0001) versus the vehicle-treated colitic group for colon length.

Vehicle-treated colitic mice progressively developed clinical signs of colitis following DSS 3% ingestion, as evidenced by increased disease activity index (DAI) scores throughout the experimental period compared to the Naive group (Figure 1A–C). The increase in DAI became more pronounced from day 4 onward and remained elevated until the end of the protocol, reflecting the progression of intestinal inflammation. During the 13-day treatment period, colitic animals treated with vehicle exhibited a mean DAI of 5.0 ± 0.8. Treatment with ART-C at 0.3, 1, and 3 mg/kg reduced the average DAI to 3.2 ± 0.4, 3.2 ± 0.5, and 2.4 ± 0.4, respectively (Figure 1A). BAC administration at the same doses resulted in mean scores of 2.9 ± 0.5, 3.5 ± 0.6, and 3.1 ± 0.6 (Figure 1B). DRU treatment yielded mean DAI scores of 3.8 ± 0.6, 3.1 ± 0.6, and 2.3 ± 0.4 respectively for 0.3, 1, and 3 mg/kg (Figure 1C). On the final day of treatment, the vehicle group reached a mean DAI of 8.3 ± 1 (*p* < 0.001 compared to non-colitic Naïve group). In contrast, ART-C reduced DAI by 35% (*p* < 0.05), 32% (*p* < 0.05), and 45% (*p* < 0.001) at 0.3, 1, and 3 mg/kg, respectively; BAC reduced DAI by 42% (*p* < 0.01), 34% (*p* < 0.05), for 0.3 and 34% (*p* = 0.0570) at 1 mg/kg respectively; and DRU reduced DAI by 24% (*p* < 0.05), 32.5% (*p* < 0.01),and 44.5% (*p* < 0.0001), at 0.3, 1 and 3 mg/kg respectively.

Colon length macroscopic analysis showed that DSS administration induced marked colon shortening in vehicle-treated colitic mice compared to Naive animals (*p* < 0.05; Figure 1D–F). Treatment with ART-C, BAC, and DRU preserved colon length relative to the vehicle group. ART-C treatment restored colon length at all tested doses, with values approaching those observed in non-colitic animals (Figure 1D). BAC produced similar protective effects, particularly at 1 and 3 mg/kg (Figure 1E). Likewise, DRU administration significantly prevented DSS-induced colon shortening across all tested doses, with the most pronounced preservation observed at 3 mg/kg (Figure 1F).

### 2.2. Histopathological Analysis Confirms Colonic Damage and Protective Effects of Treatments

**Figure 2:**
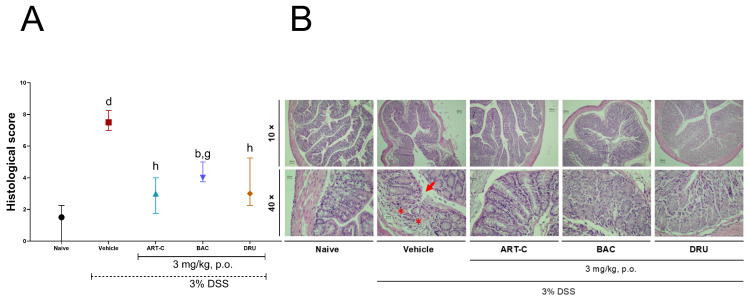
Effects of p-Coumaric acid derivatives on histological damage in DSS-induced colitic mice. (**A**) Histological scores of colonic tissue from non-colitic (Naive), vehicle-treated colitic, and treated groups with Artepillin C (ART-C), Baccharin (BAC), and Drupanin (DRU) (3 mg/kg, v.o.) following DSS (3%) administration. Mice received treatment with Vehicle (1% Tween 80 + water), ART-C, BAC, or DRU (3 mg/kg, p.o.) concomitantly with dextran sulfate sodium (DSS, 3%) administration, starting at the onset of the colitis induction protocol and continuing for 13 consecutive days. Histological scoring was based on epithelial damage, edema, inflammatory cell infiltration, goblet cell depletion, and crypt architecture. (**B**) Representative hematoxylin and eosin (H&E)-stained colon sections from each group. Upper panels show low magnification (10×), and lower panels show higher magnification (40×). The vehicle group exhibited epithelial disruption (arrow), inflammatory cell infiltration (*), and crypt damage compared to the Naive group. Treatments attenuated these histopathological alterations and preserved tissue architecture. Scale bars: 50 µm. Data in (**A**) are expressed as mean ± SEM. Statistical analysis was performed using one-way ANOVA followed by Tukey’s post hoc test. Letters indicate statistically significant differences. Letters b and d represent comparisons versus the Naive (non-colitic) group, and letters g–h represent comparisons versus the vehicle-treated colitic group: b, g (*p* < 0.001); d, h (*p* < 0.0001).

Vehicle-treated colitic mice exhibited a marked increase in histological score (7.6 ± 0.33) compared to the non-colitic (Naive) group (1.3 ± 0.49; *p* < 0.0001). Treatment with p-Coumaric acid derivatives reduced the score mean of tissue damage in 2.8 ± 0.47 (*p* < 0.0001), 4.1 ± 0.30 (*p* < 0.001) and 3.3 ± 0.84 (*p* < 0.0001) for Artepillin C (ART-C), Baccharin (BAC), and Drupanin (DRU), respectively (Figure 2A). Vehicle-treated colitic mice exhibited clear disruption of tissue organization compared to the non-colitic (Naive) group. DSS administration resulted in reduced villus height and crypt depth, as indicated by asterisks (Figure 2B). In addition, pronounced edema and inflammatory cell infiltration were observed, as highlighted by the arrow in the vehicle group. Treatment with p-Coumaric acid derivatives (3 mg/kg) partially preserved colonic structure, with improved crypt organization and reduced histopathological alterations. These effects were more evident in the ART-C and DRU-treated groups.

### 2.3. Behavioral Outcomes Following DSS-Induced Colitis and Treatment with p-Coumaric Acid Derivatives

#### 2.3.1. p-Coumaric Acid Derivatives Mitigate DSS-Induced Cognitive Deficits

**Figure 3:**
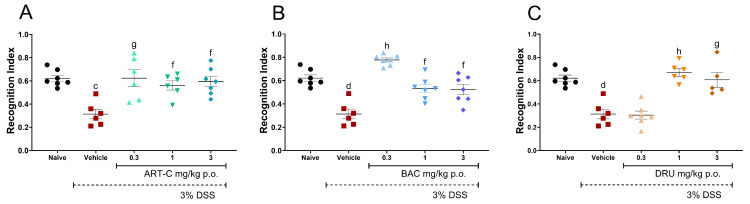
Effects of p-Coumaric acid derivatives on recognition memory in DSS-induced colitic mice. Recognition index was evaluated using the novel object recognition test. (**A**) Treatment with Artepillin C (ART-C; 0.3, 1, and 3 mg/kg, p.o.); (**B**) Baccharin (BAC; 0.3, 1, and 3 mg/kg, p.o.) and (**C**) Drupanin (DRU; 0.3, 1, and 3 mg/kg, p.o.). Mice received treatment with vehicle (1% Tween 80 + water), ART-C, BAC, or DRU (3 mg/kg, p.o.) concomitantly with dextran sulfate sodium (DSS, 3%) administration, starting at the onset of the colitis induction protocol and continuing for 13 consecutive days. Data are expressed as mean ± SEM with individual values. Statistical analysis was performed by one-way ANOVA followed by Tukey’s post hoc test. Letters indicate statistically significant differences. Letters c–d represent comparisons versus the Naive (non-colitic) group, and letters f–h represent comparisons versus the vehicle-treated colitic group: f (*p* < 0.01); c, g (*p* < 0.001); d, h (*p* < 0.0001).

The vehicle-treated group showed a 42.24 ± 10.4% (*p* < 0.001) reduction in the recognition index compared to the non-colitic naive group, as demonstrated in Figure 3. Treatment with ART-C (0.3, 1, or 3 mg/kg) reversed this effect, increasing the recognition index to 59.30 ± 32.0% (*p* < 0.001), 43.70 ± 27.20% (*p* < 0.01), and 65.40 ± 23.9% (*p* < 0.01), respectively, compared to the vehicle group (Figure 3A). Similarly, BAC administration enhanced recognition performance, with recognition indices of 116.2 ± 26.2% (*p* < 0.0001), 48.0 ± 24.2% (*p* < 0.01), and 45.5 ± 25.5% (*p* < 0.01), for 0.3, 1 and 3 mg/kg respectively, relative to vehicle-treated colitic mice (Figure 3B). Finally, DRU treatment improved recognition memory at 1 and 3 mg/kg, but not at 0.3 mg/kg, with recognition indices of 74.7 ± 31.1% (*p* < 0.0001) and 37.0 ± 33.3% (*p* < 0.001), respectively, when compared to the colitic-vehicle group (Figure 3C).

#### 2.3.2. Protective Effects of ART-C, BAC, and DRU Against DSS-Induced Anxiety-like Responses

**Figure 4:**
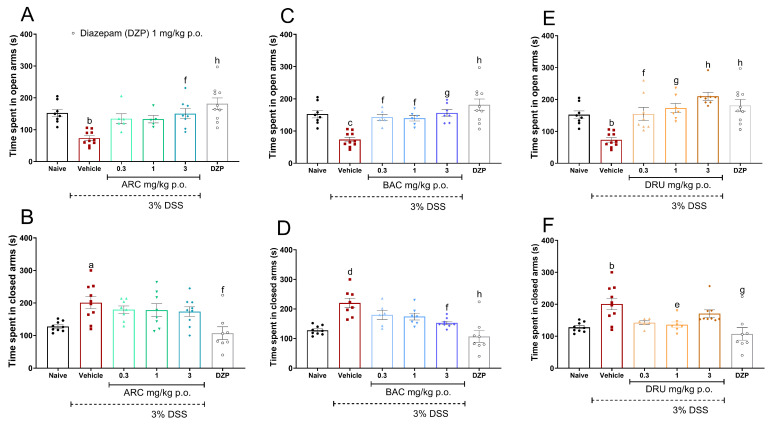
Effects of p-Coumaric acid derivatives on anxiety-like behavior in DSS-induced colitic mice assessed in the elevated plus maze. Time spent in the open arms (**A**,**C**,**E**) and closed arms (**B**,**D**,**F**) was evaluated following treatment with 0.3, 1 or 3 mg/kg, p.o of Artepillin C (ART-C), as shown in panels (**A**,**B**); Baccharin (BAC) exhibited in panels (**C**,**D**), and Drupanin (DRU) demonstrated in panels (**E**,**F**) in mice exposed to DSS (3%). Mice received treatment with Vehicle (1% Tween 80 + water), ART-C, BAC, or DRU (0.3, 1 or 3 mg/kg, p.o.) concomitantly with dextran sulfate sodium (DSS, 3%) administration, starting at the onset of the colitis induction protocol and continuing for 13 consecutive days. Diazepam (DZP, 1 mg/kg p.o.) was used as positive control. Data are expressed as mean ± SEM with individual values. Statistical analysis was performed using one-way ANOVA followed by Tukey’s post hoc test. Letters indicate statistically significant differences. Letters a–d represent comparisons versus the Naive (non-colitic) group, and letters e–h represent comparisons versus the vehicle-treated colitic group: a, e (*p* < 0.05); b, f (*p* < 0.01); c, g (*p* < 0.001); d, h (*p* < 0.0001).

As shown in Figure 4, administration of 3% DSS induced anxiety-like behavior, characterized by reduced time spent in the open arms compared with the non-colitic group (*p* < 0.01 and *p* < 0.001). As expected, diazepam (1 mg/kg, positive control) increased open-arm exploration relative to the colitic vehicle group (*p* < 0.0001). Treatment with ART-C (3 mg/kg; Figure 4A) reproduced this anxiolytic profile by increasing open-arm time (*p* < 0.01). BAC administration (0.3–3 mg/kg; Figure 4B) also increased time spent in the open arms at all doses tested (*p* < 0.01 and *p* < 0.001, respectively). Similarly, DRU treatment (Figure 4C) enhanced open-arm exploration at all doses compared with the vehicle group (*p* < 0.01, *p* < 0.001 and *p* < 0.0001, respectively).

#### 2.3.3. Protective Effects of ART-C, BAC, and DRU Against DSS-Induced Depressive-like Responses

**Figure 5:**
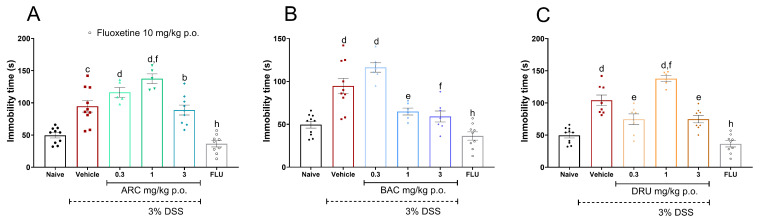
Effects of p-Coumaric acid derivatives on depressive-like behavior in DSS-induced colitic mice assessed by the tail suspension test. Immobility time was evaluated following treatment with Artepillin C (ART-C) (**A**), Baccharin (BAC) (**B**), and Drupanin (DRU) (**C**) (0.3, 1, and 3 mg/kg, p.o.) in mice exposed to DSS (3%). Mice received treatment with Vehicle (water + 1% Tween 80), ART-C, BAC, or DRU (0.3, 1 or 3 mg/kg, p.o.) concomitantly with dextran sulfate sodium (DSS, 3%) administration, starting at the onset of the colitis induction protocol and continuing for 13 consecutive days. Fluoxetine (FLU, 10 mg/kg) was used as a positive control. Statistical analysis was performed using one-way ANOVA followed by Tukey’s post hoc test. Letters indicate statistically significant differences. Letters b–d represent comparisons versus the Naive (non-colitic) group, and letters e, f and h represent comparisons versus the vehicle-treated colitic group: e (*p* < 0.05); b, f (*p* < 0.01); c (*p* < 0.001); d, h (*p* < 0.0001).

Figure 5 showed that the administration of 3% DSS increased immobility time in colitic vehicle-treated mice compared with non-colitic (Naive) controls. Treatment with ART-C further increased immobility time relative to both the Naive and vehicle groups (Figure 5A). In contrast, BAC treatment (Figure 5B) reduced immobility at doses of 1 and 3 mg/kg, whereas the 0.3 mg/kg dose increased immobility compared with the vehicle group. DRU treatment (Figure 5C) decreased immobility time at doses of 0.3 and 3 mg/kg relative to vehicle-treated colitic mice. Open-Field Analysis Shows Unchanged Locomotor and Exploratory Behavior (Figure S1).

### 2.4. DSS-Induced Disruption of Intestinal and Blood–Brain Barrier Integrity and Its Modulation by p-Coumaric Acid Derivatives

**Figure 6:**
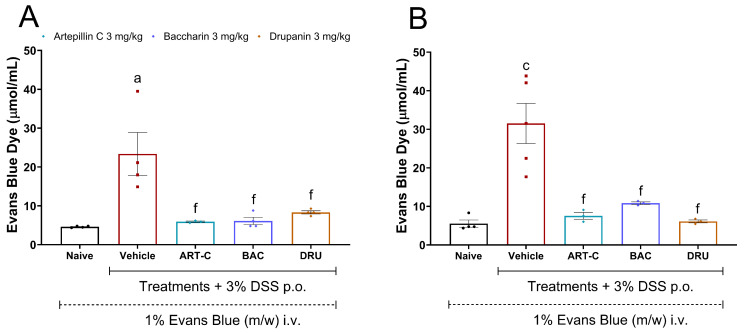
Effects of p-Coumaric acid derivatives on intestinal and blood–brain barrier permeability in DSS-induced colitic mice. (**A**) Intestinal permeability and (**B**) blood–brain barrier permeability were assessed by Evans Blue dye extravasation. Mice received treatment with Vehicle (1% Tween 80 + water), ART-C, BAC, or DRU (3 mg/kg, p.o.) concomitantly with dextran sulfate sodium (DSS, 3%) administration, starting at the onset of the colitis induction protocol and continuing for 13 consecutive days. Data are expressed as mean ± SEM (N = 3–4). Statistical analysis was performed using one-way ANOVA followed by Tukey’s post hoc test. Letters indicate statistically significant differences. Letters a and c represent comparisons versus the Naive (non-colitic) group, and letter f represents comparisons versus the vehicle-treated colitic group: a (*p* < 0.05); f (*p* < 0.01); c (*p* < 0.001).

Evans Blue dye levels measured in the intestine (Figure 6A) and the whole brain (Figure 6B) supernatant indicate biological barrier integrity. DSS administration increased intestinal permeability in vehicle-treated colitic mice compared to the Naive group (*p* < 0.05), as evidenced by elevated Evans Blue dye levels (Figure 6A). Treatment with ART-C, BAC, and DRU (3 mg/kg) significantly reduced intestinal permeability (*p* < 0.01) compared to the vehicle group, indicating preservation of epithelial barrier integrity. Similarly, as shown in Figure 6B, DSS exposure increased blood–brain barrier permeability, as demonstrated by higher Evans Blue levels in the vehicle-treated colitic group relative to Naive animals (*p* < 0.001). Treatment with p-Coumaric acid derivatives attenuated this effect, with all compounds significantly reducing dye extravasation compared to vehicle-treated colitic mice (*p* < 0.01).

### 2.5. Peripheral and Central Inflammatory Signaling in DSS-Induced Colitis and Its Modulation by p-Coumaric Acid Derivatives

#### 2.5.1. Effects of p-Coumaric Acid Derivatives on Pro-Inflammatory Cytokines in Colon of DSS-Induced Colitic Mice

**Figure 7:**
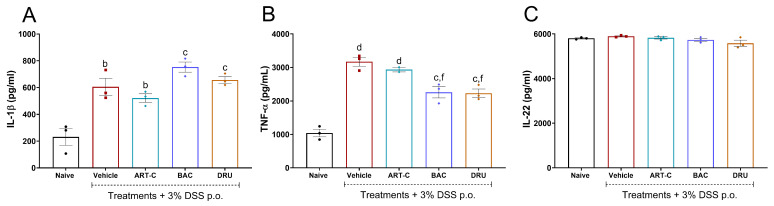
Effects of p-Coumaric acid derivatives on pro-inflammatory cytokines in colon of DSS-induced colitic mice. Levels of interleukin-1β (IL-1β; (**A**)), tumor necrosis factor-α (TNF-α; (**B**)), and interleukin-22 (IL-22; (**C**)) were quantified in colon of non-colitic (Naive), vehicle-treated colitic (1% Tween 80 + water), and colitic-treated groups with Artepillin C (ART-C), Baccharin (BAC), and Drupanin (DRU) (3 mg/kg, p.o.). Mice received treatment concomitantly with dextran sulfate sodium (DSS, 3%) administration, starting at the onset of the colitis induction protocol and continuing for 13 consecutive days. Data are expressed as mean ± SEM (N = 3–4). Statistical analysis was performed using one-way ANOVA followed by Tukey’s post hoc test. Letters indicate statistically significant differences. Letters (b, c and d) represent comparisons versus the Naive (non-colitic) group, and letter (f) represents comparisons versus the vehicle-treated colitic group: b, f (*p* < 0.01); c (*p* < 0.001); d (*p* < 0.0001).

Figure 7 demonstrated the cytokine profile for the colon. DSS administration significantly increased IL-1β and TNF-α levels (*p* < 0.01 and *p* < 0.0001, respectively, Figure 7A) of vehicle-treated colitic mice compared to the Naive group. Treatment with ART-C, BAC, and DRU 3 mg/kg did not reverse IL-1β levels, which remained elevated. Regarding TNF-α levels, treatments with p-Coumaric acid derivatives attenuated this increase in a compound-dependent manner. BAC and DRU reduced these levels compared to the vehicle-treated group (*p* < 0.05), while ART-C showed a less pronounced effect (Figure 7B).

#### 2.5.2. Effects of p-Coumaric Acid Derivatives on Pro-Inflammatory Cytokines in Serum of DSS-Induced Colitic Mice

**Figure 8:**
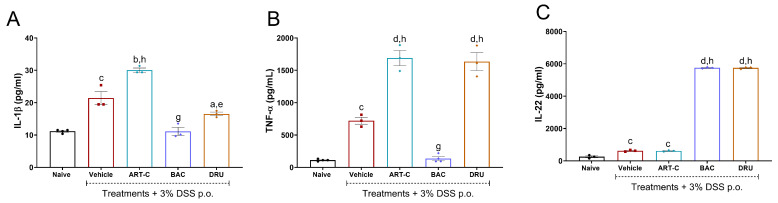
Effects of p-Coumaric acid derivatives on pro-inflammatory cytokines in serum of DSS-induced colitic mice. Levels of interleukin-1β (IL-1β; (**A**), tumor necrosis factor-α (TNF-α; (**B**), and interleukin-22 (IL-22; (**C**) were quantified in serum of non-colitic (Naive), vehicle-treated colitic (1% Tween 80 + water), and colitic-treated groups with Artepillin C (ART-C), Baccharin (BAC), and Drupanin (DRU) (3 mg/kg, p.o.). Mice received treatment concomitantly with dextran sulfate sodium (DSS, 3%) administration, starting at the onset of the colitis induction protocol and continuing for 13 consecutive days. Data are expressed as mean ± SEM (N = 3–4). Statistical analysis was performed using one-way ANOVA followed by Tukey’s post hoc test. Letters indicate statistically significant differences. Letters (a–d) represent comparisons versus the Naive (non-colitic) group, and letters (e, g and h) represent comparisons versus the vehicle-treated colitic group: a, e (*p* < 0.05); b (*p* < 0.01); c, g (*p* < 0.001); d, h (*p* < 0.0001).

Elevated IL-1β, TNF-α and IL-22 levels were also detected in the serum of vehicle-treated colitic mice compared to the Naive group (*p* < 0.001, Figure 8), which could indicate activation of the peripheral inflammatory response. In serum, ART-C further increased IL-1β levels, whereas BAC and DRU reduced this cytokine compared to the vehicle group (*p* < 0.001 and *p* < 0.05 respectively for BAC and DRU, Figure 8A). The same profile was observed for TNF-α detection, which BAC 3 mg/kg was capable of to decrease the levels compared to the vehicle-treated group (*p* < 0.001, Figure 8B). Regarding IL-22, In serum, DSS induced a modest increase in IL-22 levels (Figure 8C) comparing the vehicle-treated group to non-colitic naive mice (*p* < 0.001), which was markedly enhanced by treatment with BAC and DRU (*p* < 0.0001).

#### 2.5.3. Effects of p-Coumaric Acid Derivatives on Pro-Inflammatory Cytokines in Hippocampus of DSS-Induced Colitic Mice

**Figure 9:**
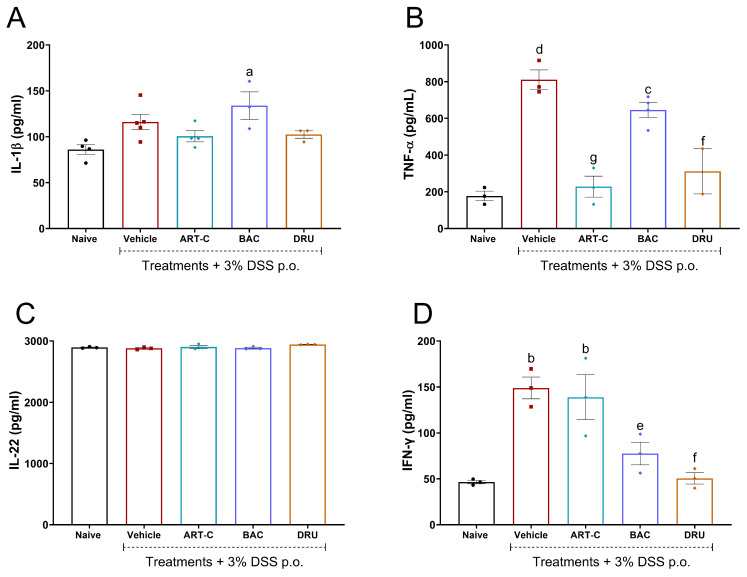
Effects of p-Coumaric acid derivatives on pro-inflammatory cytokines in hippocampus of DSS-induced colitic mice. Levels of interleukin-1β (IL-1β; (**A**)), tumor necrosis factor-α (TNF-α; (**B**)), interleukin-22 (IL-22; (**C**)) and Interferon *gamma* (IFN-γ; (**D**)) were quantified in hippocampus of non-colitic (Naive), vehicle-treated colitic (1% Tween 80 + water), and colitic-treated groups with Artepillin C (ART-C), Baccharin (BAC), and Drupanin (DRU) (3 mg/kg, p.o.). Mice received treatment concomitantly with dextran sulfate sodium (DSS, 3%) administration, starting at the onset of the colitis induction protocol and continuing for 13 consecutive days. Data are expressed as mean ± SEM (N = 3–4). Statistical analysis was performed using one-way ANOVA followed by Tukey’s post hoc test. Letters indicate statistically significant differences. Letters a–d represent comparisons versus the Naive (non-colitic) group, and letters e–g represent comparisons versus the vehicle-treated colitic group: a, e (*p* < 0.05); b, f (*p* < 0.01); c, g (*p* < 0.001); d (*p* < 0.0001).

In the hippocampus (Figure 9), ART-C markedly decreased TNF-α levels compared to vehicle-treated colitic group (*p* < 0.001) (Figure 9B). Although this effect was more evident for ART-C, treatment with BAC and DRU also altered hippocampal TNF-α concentrations compared to vehicle-treated colitic group (*p* < 0.05 and *p* < 0.01, respectively). No significant differences were observed in hippocampal IL-1β and IL-22 levels among groups (Figure 7A,C). To complement the evaluation of neuroinflammation in the hippocampus, IFN-γ levels were also assessed as an additional marker of central inflammatory signaling. Hippocampal IFN-γ levels were significantly increased following DSS administration, rising from approximately 45 pg/mL in Naive animals to 149 pg/mL in the vehicle-treated colitic group (*p* < 0.01). Treatment with BAC and DRU significantly reduced IFN-γ concentrations compared with vehicle-treated colitic mice (*p* < 0.05 and *p* < 0.01, respectively), restoring values close to those observed in the Naïve group (Figure 9D).

### 2.6. Modulation of Cholinergic Signaling by p-Coumaric Acid Derivatives in DSS-Induced Colitis

**Figure 10:**
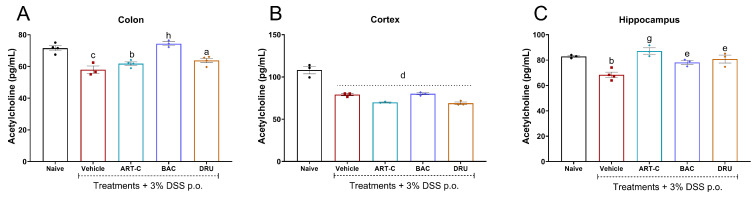
Effects of p-Coumaric acid derivatives on acetylcholine levels in colon, cortex, and hippocampus of DSS-induced colitic mice. Acetylcholine levels were measured in the (**A**) colon, (**B**) cortex, and (**C**) hippocampus of non-colitic (Naive), vehicle-treated colitic, and treated groups with Artepillin C (ART-C), Baccharin (BAC), and Drupanin (DRU) (3 mg/kg, p.o.). Treatments were administered concomitantly with dextran sulfate sodium (3% DSS) from the onset of colitis induction and maintained for 13 consecutive days. Data are expressed as mean ± SEM (N = 3–4). Statistical analysis was performed using one-way ANOVA followed by Tukey’s post hoc test. Letters indicate statistically significant differences. Letters a–d represent comparisons versus the Naive (non-colitic) group, and letters e, g and h represent comparisons versus the vehicle-treated colitic group: a, e (*p* < 0.05); b (*p* < 0.01); c, g (*p* < 0.001); d, h (*p* < 0.0001).

DSS administration significantly reduced acetylcholine levels in the colon, cortex and hippocampus of vehicle-treated colitic mice compared to the Naive group (*p* < 0.001, *p* < 0.0001 and *p* < 0.01, respectively), indicating impairment of cholinergic signaling (Figure 10A–C). In the colon, BAC significantly increased this neurotransmitter compared to the vehicle group (*p* < 0.0001; Figure 10A). In the hippocampus, ART-C markedly increased acetylcholine levels compared to the vehicle group (*p* < 0.001), restoring values close to or above those observed in Naive animals, while BAC and DRU produced moderate improvements (*p* < 0.05; Figure 10C).

### 2.7. Modulation of Inflammatory and Oxidative Stress Markers by p-Coumaric Acid Derivatives in DSS-Induced Colitis

#### 2.7.1. Oxidative Stress Biomarkers in the Colon of DSS-Induced Colitic Mice Treated with p-Coumaric Acid Derivatives

DSS administration significantly impaired antioxidant defenses, as evidenced by reduced SOD, CAT and GSH activity in the colon of vehicle-treated compared to the non-colitic group (*p* < 0.05—SOD; *p* < 0.01—CAT and GSH, Table 1). Furthermore, increased lipid peroxidation (MDA) and GST activity compared to the Naive group (*p* < 0.0001 and *p* < 0.001, respectively). p-Coumaric Acid Derivatives 3 mg/kg were capable of restored antioxidant enzyme activity, increasing SOD (*p* < 0.01 and *p* < 0.001 for ART-C, BAC and DRU, respectively), CAT (*p* < 0.000 for ART-C), and GSH levels (*p* < 0.01 and *p* < 0.0001 for ART-C, BAC and DRU, respectively), reduced oxidative damage in the colon decreasing MDA (*p* < 0.0001) and normalizing GST levels when compared to the vehicle-treated colitic group (*p* < 0.0001, *p* < 0.001 and *p* < 0.0001 for ART-C, BAC and DRU, respectively).

#### 2.7.2. Oxidative Stress Biomarkers in the Cortex and Hippocampus of DSS-Induced Colitic Mice Treated with p-Coumaric Acid Derivatives

Oxidative imbalance can also be seen in the cortex (Table 2), as a result of DSS exposure, as evidenced by a decrease in CAT and GSH activity (*p* < 0.01 and *p* < 0.0001, respectively) and increased GST (*p* < 0.01) and MDA levels (*p* < 0.0001) when comparing the vehicle-treated colitic to the non-colitic group (naive). BAC and DRU treatment markedly enhanced CAT activity (*p* < 0.001 and *p* < 0.0001, respectively) and GSH activity (*p* < 0.0001), whereas ART-C exhibited a modest modulation in GSH activity (*p* < 0.05) compared to the vehicle-treated colitic group. In parallel, treatments also promoted reduced lipid peroxidation, decreasing MDA levels (*p* < 0.001 and *p* < 0.0001 for ART-C, BAC and DRU, respectively) compared with the vehicle-treated colitic group. In the hippocampus (Table 3), DSS promoted lipidic peroxidation, evidenced by an increase in MDA levels (*p* < 0.0001) and enhanced GST activity (*p* < 0.05) compared to the non-colitic group (naive). Treatment with the p-Coumaric acid derivatives normalized MDA levels, compared to vehicle-treated colitic (*p* < 0.05 and *p* < 0.01 for ART-C, BAC and DRU, respectively).

#### 2.7.3. Inflammatory Response Modulated by p-Coumaric Acid Derivatives in DSS-Induced Colitis

**Figure 11:**
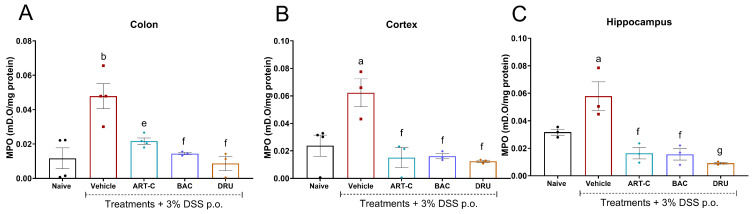
Effects of p-Coumaric acid derivatives on Myeloperoxidase (MPO) levels in colon, cortex, and hippocampus of DSS-induced colitic mice. MPO levels were measured in the (**A**) colon, (**B**) cortex, and (**C**) hippocampus of non-colitic (Naive), vehicle-treated colitic, and treated groups with Artepillin C (ART-C), Baccharin (BAC), and Drupanin (DRU) (3 mg/kg, p.o.). Treatments were administered concomitantly with dextran sulfate sodium (3% DSS) from the onset of colitis induction and maintained for 13 consecutive days. Data are expressed as mean ± SEM (N = 3–4). Statistical analysis was performed using one-way ANOVA followed by Tukey’s post hoc test. Letters indicate statistically significant differences. Letters a–b represent comparisons versus the Naive (non-colitic) group, and letters e–g represent comparisons versus the vehicle-treated colitic group: a, e (*p* < 0.05); b, f (*p* < 0.01); g (*p* < 0.001).

MPO activity was assessed as an indicator of neutrophil-mediated inflammatory responses. As shown in Figure 11, DSS administration significantly increased MPO activity in the colon (*p* < 0.01, Figure 11A), cortex (*p* < 0.05, Figure 11B), and hippocampus (*p* < 0.05, Figure 11C) compared with Naive animals, which could indicate enhanced inflammatory cell activation following experimental colitis. In colonic tissue (Figure 11A), treatment with ART-C significantly reduced MPO activity (*p* < 0.05 vs. vehicle-treated colitic group), whereas BAC and DRU produced an even greater reduction (*p* < 0.01 vs. vehicle-treated colitic group, respectively), restoring values close to those observed in non-colitic animals.

Similar findings were observed in the cortex (Figure 11B), where treatment with ART-C, BAC, and DRU significantly attenuated this increase (*p* < 0.01 vs. vehicle-treated colitic group). All tested compounds reduced MPO activity in the hippocampus (Figure 11C) compared with vehicle-treated colitic mice, with ART-C and BAC producing significant reductions (*p* < 0.01), while DRU exhibited the most pronounced effect (*p* < 0.001).

## 3. Discussion

Biological events that culminate in neurodegenerative processes may begin in peripheral sites and project to the CNS [7,14,15]. One of the hypotheses that has been gaining strength in recent years to elucidate part of the peripheral mechanisms that may contribute to neuroinflammation and neurodegeneration processes is the presence of inflammatory bowel diseases (IBDs) as precursors, which trigger systemic processes that, under certain conditions, would be capable of disrupting CNS homeostasis [16]. In this context, the data collected here strengthen the hypothesis that compounds that reduce the peripheral inflammatory damage associated with such intestinal diseases may also be promising in preventing the progression of inflammatory and/or degenerative processes in the CNS.

The compounds studied in this work are prenylated derivatives of p-Coumaric acid, Artepillin C (ART-C), Baccharin (BAC), and Drupanin (DRU), and are part of the chemical composition of Brazilian green propolis (BGP). This type of propolis has central and peripheral biological effects, notably anti-inflammatory potential [13,17], neuroprotection [11,18] and gastroprotective effect [19,20]. To investigate the ability of these compounds to attenuate experimental induced colitis and intestinal damage, thereby limiting systemic inflammation and gut–brain axis dysfunction, this study used an animal model of acute UC, widely reported in the literature, which uses oral ingestion of dextran sulfate sodium (DSS) as an inflammatory agent, allowing the screening of experimental drugs with anti-inflammatory action in the colon [21,22,23]. The UC model based on DSS ingestion is a widely used methodology in the study of IBDs, which is highly compatible with the clinical context because it reproduces in animals symptoms similar to those observed in UC crises in humans. Manifestations include bloody diarrhea, mucosal ulceration, loss of epithelial cells, and neutrophil infiltration in the gastrointestinal tract [24,25,26].

A marked weight loss was promoted in 3% DSS intake, as observed in the colitic animals (vehicle-treated). This effect of DSS administration may be related to damage to the intestinal inflammation and tissue damage, which can affect barrier function in addition to an intense inflammatory process caused by the phlogistic agent that promotes severe damage to the intestinal tunics and other histological compartments of the intestine. Metabolic changes resulting from the release of cathepsin B, a protein involved in mitochondrial dysfunction and the disruption of tight junctions (TJs), were reported in the context of myosin light chain kinase (MLCK)-induced intestinal epithelial damage [27]. Treatments mitigated the effects of disease progression, which was observed through a decrease in the score marked by the disease activity index (DAI). Furthermore, when comparing the colon length of animals in each group, the animals in the vehicle-treated group had a decrease in length compared to the animals in the naive group, indicating the reproducibility of the model and its similarity to the human disease [24,28]. This effect was also minimized by p-Coumaric acid derivatives, which showed no differences in size when compared to the naive group. Furthermore, when analyzing the activity of myeloperoxidase (MPO), an enzyme derived from leukocytes that catalyzes the formation of hypochlorous acid (HOCl) in oxidative processes, an attenuation of its levels was observed not only in the colon (which is consistent with histological findings) but also in the cortex and hippocampus of colitic animals treated with p-Coumaric acid derivatives. This provides unprecedented evidence of the effect of ART-C, BAC, and DRU compounds on this enzymatic activity.

In this regard, investigating changes in colonic permeability and possible alterations in the Intestinal–Epithelial Barrier of animals with 3% DSS-induced colitis using intravenous administration of dyes is a practical tool for indirectly demonstrating how intestinal contents enter the systemic circulation, since these are high-molecular-weight compounds (>180 Da) that are unable to permeate an intact barrier. Changes in intestinal permeability in the 3% DSS model using the intracolonic EB administration technique have already been reported by Bai et al. (2022) [29], who observed increased intestinal permeability in pathogen-free C57BL/6J mice and attributed this finding to decreased expression of proteins such as occludins, ZO-1, and E-cadherin, which play an important role in the formation and maintenance of intestinal barrier tight junctions. In the present study, through intravenous administration of the EB dye, not only was an increase in intestinal permeability observed, but an increase in BBB permeability—indicated by the increased concentration of the dye in the brains collected from these animals—was also visualized for the first time using this colitis induction model.

Although a more accurate assessment is needed using other techniques, such as fluorescein isothiocyanate-dextran (FITC-d) for intestinal barrier evaluation, for example, it can be inferred that the 3% DSS-induced colitis model causes significant changes in membrane permeability, particularly at the blood–brain barrier (BBB). The hypothesis that intestinal damage facilitates the release of intestinal contents, or even metabolic byproducts resulting from the heightened inflammatory process, suggests that this process may trigger a systemic response that affects the integrity of the BBB, thereby leading to the observed behavioral responses.

Considering that the research hypothesis was to establish a link between the changes caused by the intestinal inflammatory process and behavioral changes resulting from the neuroinflammation process, one of the important points of the work carried out was to verify the influence that the model of inflammatory bowel disease could have on memory and, consequently, play a significant role in the progression of neuroinflammation. A decrease in IR was evidenced by comparing colitic vehicle-treated to non-colitic animals, suggesting that the disease state of acute colitis can promote recognition memory impairments in these animals, where the animal is unable to distinguish between familiar and new objects. The results also demonstrate that treatments with ART-C, BAC, and DRU promoted an increase in IR in these animals when compared to the colitic group (vehicle group), causing this group to spend more time exploring the new object, indicating that the treatment can alleviate the cognitive deficits induced by DSS. The model of colitis caused by DSS ingestion in C57BL/6 mice promotes less exploration of the new object, implying a decrease in IR, which translates into cognitive decline [30], which is consistent with those presented in the current study. In addition, the deleterious effects of DSS on the memory of animals evaluated in the Morris water maze have also been described [31], and finally, aversive memory impairment in animals subjected to the inhibitory avoidance test. In this sense, the data together indicate that not only is the colitis model itself capable of inducing memory impairment, but it also interferes with different types of memory.

It has been reported that systemic inflammation induced by colitis may be an important contributor to the onset of anxiety and depression [32,33,34,35]. However, there is little literature demonstrating this relationship in animal models. In order to establish this relationship, it was proposed to evaluate the possible anxiety-like behavior of animals treated with DSS3% and the anxiolytic-like effect of the compounds under study, using the elevated plus maze (EPM). The state of illness induced by ingestion of 3% DSS promoted distinct behavioral changes, observed in the EPM, where the colitic animals remained longer in the closed arms of the apparatus, compared to the non-colitic group and the group administered the anxiolytic diazepam. The results obtained on the effects of DSS on anxiety-like behavior are consistent with previous findings that demonstrated a reduction in open arm entry in animals with induced colitis [36,37,38]. These findings reinforce that DSS treatment increases anxiety-like behavior in mice. Furthermore, in the present study, anxious-like behavior was reversed by treatment with ART-C, BAC, and DRU, especially at doses of 3 mg/kg. In this context as well, the literature reports that intracerebral injections of lipopolysaccharide (LPS) induce anxiety-like behavior in animals [39], increasing the time spent in closed arms in the LCE. In addition, changes in the composition of the microbiota (dysbiosis with increased LPS concentrations) have also been reported to induce anxiogenic effects in the LCE [40]. In this sense, hypotheses are conducted within the UC model by DSS regarding the disruption of the intestinal barrier induced by the agent, which may be capable of promoting the arrival of endotoxins via the systemic route to the CNS, causing the same effect observed in an i.c.v. injection of LPS, and promoting anxiety-related behaviors, as observed clinically.

Alterations in the barriers may exacerbate the imbalance in the redox state of the systemic and cerebral microenvironment [7,41]. In this context, investigating possible systemic changes by measuring certain markers of the inflammatory process in the animals’ serum may provide a pathway to explain the molecular process of interactions with the membranes. The detection of certain concentrations of pro-inflammatory cytokines such as IL-1β, TNF-α and IL-22, not only systemically but also in specific organs, is an important marker of inflammation progression and has been extensively implicated in the disruption of intestinal barrier integrity, amplification of local inflammatory responses, and propagation of systemic inflammation.

A clinical study conducted by Rawat et al. (2020) [42] identified increased concentrations of IL-1β in colon samples from biopsies of patients with UC and investigated its role in the disruption of intestinal TJs. The authors found that increased IL-1β promotes a decrease in the expression of occludins by enterocytes and consequently increases TJ permeability. The presence of IL-1β negatively interacts with proteins that form intestinal tight junctions, thereby increasing their permeability. Tracking this marker by measuring its systemic levels in serum may indicate that this cytokine could potentially cause changes in barrier permeability or even induce a process of neuroinflammation. In this study, an increase in this cytokine was identified in the serum of animals fed 3% DSS, which was attenuated by treatment with BAC and DRU, but not ART-C. Using an air-sac inflammation model, Ferreira et al. (2021) [43] demonstrated the anti-inflammatory effect of BAC through the reduction of IL-1β levels in the inflammatory process. In the present study, an increase in this cytokine was identified in the hippocampus region; however, the doses used for the experimental treatment did not influence the reduction of IL-1β levels in this region.

Unlike IL-1β, IL-22 plays an ambiguous role in the inflammatory process. This cytokine is secreted in the colon by intestinal immune cells, specifically type 3 innate lymphoid cells (ILC3s), which utilize the activation of aryl hydrocarbon receptors (AHRs) as a trigger for its synthesis [44]. In this sense, it is responsible for regulating innate immune defense, modulating cell differentiation, promoting proliferation and having important functions in colitis recovery, reinforcing mucosal barrier integrity by inducing the production of antimicrobial peptides and chemokines in different tissues [45,46]. However, it also promotes effects related to the pathogenesis of autoimmune diseases such as psoriasis [47]. It has been reported that IL-22 expression in colon biopsies from patients with severe UC is significantly higher than in patients without UC [48], and the beneficial effect of IL-22 expression has also been reported [49], demonstrating an increase in IL-22 expression related to intestinal epithelial repair, enhancing barrier integrity via AHR receptors in studies using a DSS-induced colitis model. In a study using lipidic nanoparticles encapsulated with IL-22-mRNA (IL-22/nLNPs) in experimental treatment of a model of acute colitis, mice fed with IL-22/nLNPs experienced an accelerated healing process, as indicated by the recovery of more body weight and colon length as well as reduction of the histological index, colonic MPO activity [50].

TNF-α, a key pro-inflammatory cytokine implicated in the pathogenesis of inflammatory bowel disease and gut–brain axis dysfunction, was markedly increased in the colon, serum, and hippocampus following DSS administration, supporting the establishment of a robust inflammatory state extending beyond the intestinal compartment. The elevation of this circulating cytokine is particularly relevant because it can influence blood–brain barrier permeability and promote neuroinflammatory signaling, thereby contributing to inflammatory alterations in brain regions associated with cognition and behavior, including the hippocampus [51,52,53]. Treatment with the p-coumaric acid derivatives attenuated TNF-α levels in a tissue-dependent manner.

In the colon, BAC and DRU significantly reduced TNF-α concentrations, suggesting an ability to modulate local inflammatory pathways activated during DSS-induced mucosal injury. Interestingly, BAC also reduced serum TNF-α levels, indicating a broader systemic anti-inflammatory effect. In contrast, ART-C exerted its most pronounced action in the hippocampus, where it significantly decreased TNF-α levels, suggesting a potential neuroprotective effect against inflammation-driven alterations within the central nervous system. These findings indicate that although all three compounds exhibited anti-inflammatory properties, their effects were not identical across tissues, suggesting distinct pharmacodynamic profiles. Moreover, the anti-inflammatory effects observed for TNF-α in distinct peripheral and central tissues were accompanied by improvements in barrier integrity, oxidative stress parameters, and histopathological alterations, supporting inflammatory modulation as one of the mechanisms underlying the protective effects of these compounds.

Beyond TNF-α, microglia-related inflammatory mediators may also contribute to the propagation of neuroinflammatory responses and behavioral alterations associated with experimental colitis. IFN-γ is recognized as a potent activator of microglial cells and has been implicated in the amplification of neuroinflammatory responses and behavioral dysfunction [54,55]. The increase in hippocampal IFN-γ observed following DSS administration, together with elevated TNF-α levels and oxidative stress markers, supports the occurrence of central inflammatory alterations in this model. The reduction of IFN-γ induced by BAC and DRU suggests that these compounds may attenuate neuroinflammatory signaling pathways commonly associated with microglial activation [56], although direct assessment of microglial markers was beyond the scope of the present study.

Complementing the role of inflammatory mediators, neurotransmitter signaling represents a major component of gut–brain communication. In addition to cytokine-mediated pathways, neural mechanisms enable intestinal inflammation to influence central nervous system function and behavior. Among the neurochemical mediators involved in this bidirectional crosstalk, acetylcholine has attracted particular attention due to its well-established roles in regulating intestinal homeostasis, immune responses, and neuroinflammatory processes [57,58]. It is believed that a cholinergic anti-inflammatory pathway, mediated by this neurotransmitter, has an immunomodulatory effect, attenuating inflammation and promoting immune tolerance in the intestinal environment [59,60]. ACh modulation may be related to the induction of IL-10 secretion by monocyte-derived myeloid suppressor cells (M-MDSCs), which can lead to the activation of the extracellular signal-regulated kinase (ERK) pathway, resulting in an immunoregulatory response and exerting the anti-inflammatory effects of interleukin [61]. Dysregulation of this pathway may contribute to the development or exacerbation of IBD [62,63].

In addition to its immunoregulatory role in the enteric nervous system, ACh is an important neurotransmitter in the CNS that performs various cognitive functions, particularly those related to memory encoding and consolidation [64]. Its release in the hippocampus, a brain region that plays a fundamental role in memory, is associated with the modulation of synaptic plasticity, which refers to the ability of synapses to strengthen and reorganize over time—a crucial process in learning and memory, which involves increasing synaptic transmission in the hippocampus, facilitating the encoding of new information into long-term memory [65]. Thus, disruptions in the cholinergic system have been implicated in cognitive disorders, such as AD [64].

Originally, the present study demonstrated that the acute UC model promoted deleterious effects on ACh levels, primarily in the cortex and hippocampus of colitis-induced animals compared to non-colitis animals. The reduction in the neurotransmitter reached 26.8% compared to non-colitic animals. Although the p-Coumaric acid derivatives did not consistently restore acetylcholine levels across all evaluated tissues, selective cholinergic modulation was observed. Baccharin significantly increased acetylcholine levels in the colon, whereas Artepillin C enhanced acetylcholine concentrations in the hippocampus. These findings suggest a tissue-dependent effect of these compounds on cholinergic signaling.

In parallel with alterations in cholinergic signaling, oxidative stress has emerged as a major contributor to the intestinal and neurological disturbances associated with DSS-induced colitis. The interplay between inflammatory mediators, neurotransmitter systems, and redox imbalance is increasingly recognized as a critical component of gut–brain axis dysfunction. Due to its elevated oxygen consumption and intense mitochondrial activity, the brain is particularly susceptible to oxidative imbalance and ROS-mediated damage ([66]). In this context, antioxidant enzymes such as superoxide dismutase (SOD), catalase (CAT), reduced glutathione (GSH), and glutathione S-transferase (GST) play essential roles in maintaining redox homeostasis and protecting tissues against oxidative injury.

Consistent with previous reports in DSS-induced colitis models [21,67], the present study demonstrated a reduction in SOD activity in colonic tissue from colitic animals, which was reversed mainly by treatment with the p-Coumaric acid derivatives at doses of 1 and 3 mg/kg. Similar modulation was observed in cortical and hippocampal tissues, suggesting that these compounds exert antioxidant effects not only locally in the intestine but also in extra-intestinal regions susceptible to systemic inflammatory damage. Previous studies with Brazilian green propolis extracts and isolated Artepillin C, Baccharin, and Drupanin also reported restoration of antioxidant enzyme activity [19,20], supporting the hypothesis that these compounds may interact with endogenous antioxidant defense systems. Mechanistically, the induction of SOD activity may involve preservation of enzymatic function under oxidative conditions, modulation of transcriptional pathways, and activation of antioxidant signaling pathways such as Nrf2/ARE [68], which are recognized therapeutic targets in both inflammatory bowel disease and Alzheimer’s disease.

Additionally, DSS exposure reduced GSH levels in the colon and cortex, whereas treatment with the p-Coumaric acid derivatives, particularly at 3 mg/kg, restored these levels. Since GSH and GST act synergistically in cellular detoxification and free radical scavenging, restoration of these antioxidant systems reinforces the protective effects of the compounds against DSS-induced oxidative damage. CAT activity was also reduced in colitic animals and normalized following treatment. Collectively, these findings indicate that modulation of endogenous antioxidant defenses may represent an important mechanism underlying the protective effects of these compounds in intestinal and central tissues.

Oxidative stress is closely associated with lipid peroxidation, a process in which ROS damage membrane lipids and generate toxic secondary products such as malondialdehyde (MDA), a well-established marker of oxidative injury. Elevated MDA levels have been implicated in neuroinflammatory and neurodegenerative conditions, including Alzheimer’s disease and Parkinson’s disease [69]. In the present study, DSS administration increased MDA levels in the colon, cortex, and hippocampus, indicating that intestinal inflammation induced systemic oxidative damage extending to brain regions associated with cognition and neuroinflammation. Importantly, treatment with the p-coumaric acid derivatives at 3 mg/kg significantly reduced MDA levels in all evaluated tissues, suggesting attenuation of lipid peroxidation and a potential neuroprotective effect against DSS-induced oxidative and inflammatory insults.

The present study provides evidence that prenylated p-Coumaric acid derivatives exert beneficial effects on both intestinal and central alterations associated with DSS-induced colitis. The observed improvements in disease activity, intestinal barrier integrity, inflammatory signaling, oxidative stress parameters, and behavioral outcomes support the hypothesis that modulation of gut–brain axis dysfunction contributes to the protective effects of these compounds.

Nevertheless, this study has limitations. Although the evaluation of inflammatory cytokines, oxidative stress biomarkers, and acetylcholine levels provided important insights into the mechanisms underlying the observed behavioral improvements, direct assessment of key cellular mediators of neuroinflammation was not performed. In particular, microglial activation, which represents a critical link between peripheral inflammation and central nervous system dysfunction, was not directly investigated through specific markers such as Iba-1 or CD68. Likewise, while reductions in hippocampal TNF-α and IFN-γ levels support attenuation of neuroinflammatory signaling, the causal relationship between these molecular alterations and the behavioral effects observed in the present study requires further validation. Future studies employing immunohistochemical, molecular, and functional approaches will be important to clarify the contribution of microglial activation, cholinergic modulation, and other neuroimmune pathways to the neuroprotective effects of these prenylated p-coumaric acid derivatives. Such investigations may further establish these compounds as promising candidates for the management of intestinal inflammation and its associated gut–brain axis dysfunction.

## 4. Materials and Methods

### 4.1. Chemicals

Dextran sulfate sodium (DSS) with a molar weight of ~40,000 was purchased from Sigma-Aldrich^®^ (Cat. No. 42867). The p-Coumaric acid derivatives were obtained from the crude extract of Brazilian green propolis, as described previously, using hexane and ethyl acetate in gradient elution [70]. Subsequently, the subfractions rich in these compounds were purified by High Performance Liquid Chromatography (HPLC-UV) using a Shim-pack Prep-ODS column (15 µm, 20 × 250 mm; Shimadzu Corporation, Kyoto, Japan), yielding pure Artepillin C (1.68 g), Drupanin (0.41 g), and Baccharin (0.67 g), with a relative purity of 97% by HPLC-DAD at 275 nm. The isolated compounds Artepillin C, Baccharin, and Drupanin were identified by spectroscopic (1H NMR and 13C NMR) and spectrometric (high-resolution ESI-MS) analyses, in comparison with literature data [70,71].

### 4.2. Animals

Female Swiss mice (*Mus musculus*) weighing 25–35 g, approximately 3 months old, were used. They were obtained from the central animal facility at UNIVALI, where they were kept under controlled conditions of temperature (22 ± 2 °C) and a 12:12 h light/dark cycle, with free access to water and food. Individual mice were used as the analytical unit for behavioral, biochemical, and histological outcomes. All pharmacological tests were performed according to the National Council for the Control of Animal Experimentation (CONCEA), with the experimental protocol previously approved by the Ethics Committee for Animal Use (CEUA), obtaining approval protocol No. 029/22.

The animals were randomly separated into groups (five per cage) according to the experimental treatments using p-Coumaric acid derivatives *per oral*: Artepillin (ART-C), Baccharin (BAC), or Drupanin (DRU) at doses of 0.3, 1, or 3 mg/kg, and a negative control represented by the vehicle group (Distilled water + Tween 1% 0.1 mL/10 g). The sample size and doses chosen for the treatments were based on previous studies [19,20,21]. Behavioral, histological, and biochemical analyses were performed by investigators blinded to treatment allocation.

### 4.3. Acute Colitis Protocol Using 3% Dextran Sulfate Sodium (DSS) Ingestion

The animals were subjected to the acute colitis protocol by ingesting 3% (weight/volume) DSS (MM: 40,000) dissolved in drinking water for seven days [21,22,28]. DSS solution was administered through shared drinking bottles at the cage level, whereas pharmacological treatments were administered individually by oral gavage, where treatment with the compounds and controls described above began concurrently with DSS ingestion and lasted for 13 days. From the first day of DSS administration concomitant with treatments with p-Coumaric acid derivatives (0.3, 1, or 3 mg/kg), the animals were individually weighed and evaluated for rectal bleeding and stool consistency daily, with these evaluations preferably performed in the morning by the same observer. Each parameter received a score that was recorded and used to calculate the disease activity index (DAI), indicative of colitis progression and used to assess treatment efficacy [72]. Animals presenting severe clinical deterioration or unexpected mortality were excluded.

### 4.4. Evaluation of the Effect of p-Coumaric Acid Derivatives on the Behavior of Animals with UC Inducted with 3% DSS Ingestion

On the 8th day after the start of DSS administration and treatment with p-Coumaric acid derivatives, the animals underwent different behavioral tests to assess parameters related to locomotion, recognition memory, anxiety-like behavior, and depression-like behavior. The open field test is widely used to assess locomotory activity and exploration habits [73]. The apparatus consists of a box measuring 30 × 30 × 30 cm with white walls and floor divided into nine identical quadrants. Each animal was placed individually in the central quadrant of the arena, where it was allowed to freely explore the environment for six minutes. During this time, the number of crossings, which consist of crossing all four paws per quadrant, and the number of exploratory activities, which consist of the animal standing on its hind legs, were counted.

The Novel Object Recognition assesses declarative memory in rodents, which is based on the animal’s natural tendency to explore a new object more than a familiar one, within a known context [74]. The same open field arena described above was used, with the test divided into two stages. The first moment occurred 24 h after this exposure to the apparatus, when the animal was again introduced into the Open Field environment. However, two identical objects were placed diagonally on the floor of the apparatus (objects A + A), which the animals explored for ten minutes. The second moment of the test took place the day after, when one of the objects was replaced by another that was completely different in color, size, and shape (objects A + B). The interaction time with each object was counted and recorded for ten minutes, a parameter that was later used to calculate the recognition index (RI) using the formula: object B time divided by the sum of object A and B [TB/(A + B)] [75].

Animals exposed to DSS were assessed for anxiety-like behavior in the Elevated Plus Maze apparatus, which consists of a Greek cross-shaped maze with two closed arms and two open arms (all measuring 6 × 30 cm), raised 50 cm above the floor. The test is based on the natural aversion of rodents to open environments, in which they cannot perform thigmotaxis [76,77]. The mice were placed individually in the central part of the maze and allowed to explore the apparatus for 6 min. The number of entries and the time spent on each type of arm (open or closed) were recorded. Increased exploration in the open arms reflects an anxiolytic effect of the substance being tested [78].

In the Tail Suspension Test, the animals were exposed to a wooden apparatus consisting of an aluminum bar inserted between four side compartments with acoustic and visual separation. The animals, isolated from each other, are suspended by their tails with appropriate adhesive tape, about 100 cm from the floor of the apparatus, in a position that prevents escape or support on nearby surfaces. During the six-minute test, the animal’s immobility time is counted, which is interpreted as giving up escaping the imposed aversive situation, and is used as a parameter to determine depressive-type behavior [79].

### 4.5. Assessment of Intestinal Vascular Permeability and Blood–Brain Barrier Permeability

Vascular permeability in the colon and blood–brain barrier (BBB) integrity was assessed by measuring dye extravasation after intravenous administration of Evans Blue dye [80]. The animals were properly anesthetized with xylazine (10 mg/kg, i. p.) + ketamine (50 mg/kg, i. p.) and after total loss of postural reflexes, a 1% (*w*/*v*) solution of Evans Blue (EB) prepared in saline solution was injected systemically (intravenously) through the tail vein, respecting the maximum volume to be injected according to the weight of each animal (1 mL/kg). After administration of the dye, the animals were placed in boxes to control hypothermia induced using an anesthetic, under yellow light and a thermal blanket, where they remained at rest for two hours. Subsequently, the animals were euthanized via cervical dislocation. Their brains and colons were removed and incubated in a solution of formamide (2 mL/g tissue) for 24h [81]. The supernatant of this solution was distributed into 96-well plates, and the absorbance of the liquid was measured at 620 nm, with results expressed in μmol of Evans Blue/g tissue calculated based on the Evans Blue color coefficient = 7.810/M/cm [80,82].

### 4.6. Evaluation of Oxidative Stress, Antioxidant Defense Markers and Pro-Inflammatory Cytokines

After completion of the pharmacological tests (13 days after the start of treatment and administration of DSS), the animals were euthanized by cardiac puncture followed by cervical dislocation. Immediately after the procedure, the brain was removed, and 1 mL of whole blood was stored. Homogenates were prepared from colon, cortex and hippocampus in 200 mM potassium phosphate buffer solution (pH 6.5) for quantification of reduced glutathione (GSH) [83] and malondialdehyde (MDA) [84] levels and quantification of cytokines (IL-1β, TNF-α and IL-22).

Another part of this prepared homogenate was centrifuged at 11,000 rpm for 20 min at 4 °C to obtain the supernatant used to determine the activity of superoxide dismutase (SOD) [85], glutathione s-transferase (GST) [86], and catalase (CAT) [87], and the pellet was resuspended to determine the activity of myeloperoxidase (MPO) [88,89]. For a detailed description of the methods, see Da Silva et al. (2015) [90].

The protein concentration in the tissue was determined using the Bradford method (Bio-Rad, Hercules, CA, USA), employing bovine serum albumin as a standard (0.125 to 1.0 mg/mL), according to the manufacturer’s guidelines [91].

#### Enzyme-Linked Immunosorbent Assay (ELISA) for Quantification of Cytokines and Acetylcholine (ACh)

The cytokines IL-1β, IL-22, and TNF-α were quantified in the colon, serum, and hippocampus tissues of the animals, and IFN-γ was quantified in the hippocampus tissue. All analyses followed the dilution, preparation, and reading guidelines provided by the manufacturer, using the Sigma-Aldrich^®^ ELISA kit. Absorbance was recorded at 450 nm, and individual values were interpolated on a standard curve ranging from 2.74 to 2000 pg/mL.

For the analysis of acetylcholine levels, this neurotransmitter was quantified in colon and hippocampus tissues, according to the dilution, preparation, and reading instructions recommended by the manufacturer, using the ABCAM^®^ ELISA kit. Absorbance was measured at 450 nm, and individual values were interpolated on a standard curve ranging from 5.625 to 90 pg/mL, expressed in pg/mL.

### 4.7. Histological Analysis

A 0.5 cm segment was fixed in a solution composed of 85% alcohol, 10% formaldehyde, and 5% acetic acid. Subsequently, the samples underwent dehydration, clearing, and paraffin embedding. Sections of 5 μm were then deparaffinized and stained with eosin-hematoxylin for morphological analysis. Parameters observed generated a score according to (1) mucosal epithelium: 0—intact epithelial surface; 1—loss of <5% of the epithelial surface; 2—loss of 5–10% of the epithelial surface; and 3—loss of >10% of the epithelial surface. According to (2) crypt condition: 0—intact; 1—loss of <10% of crypts; 2—loss of 10–20% of crypts; and 3—loss of >20% of crypts, and finally according to (3) cellular infiltration and edema: 0—none; 1—mild; 2—moderate; and 3—severe [21,72].

### 4.8. Statistical Analysis

The results were presented as means, followed by the respective mean standard errors (SEM), and submitted to one-way or two-way analysis of variance (ANOVA), as necessary. When applicable, multiple comparison tests were performed using Dunnett’s and Tukey’s post hoc models. Effect magnitude was interpreted based on differences between group means and percentage changes relative to control groups, together with mean ± SEM values. The evaluation of nonparametric tests, the Kruskal-Wallis test was used, followed by Dunn’s post-test, using GraphPad Prism^®^ software version 8.0. Values of *p* < 0.05 were considered statistically significant.

## 5. Conclusions

Collectively, the present findings demonstrate that DSS-induced colitis promotes not only severe intestinal inflammation, but also systemic and central alterations associated with neuroinflammatory and neuropsychiatric impairments, reinforcing the concept of a functional microbiota–gut–brain axis in chronic inflammatory conditions. In this context, the major prenylated p-Coumaric acid derivatives from Brazilian green propolis, Artepillin C, Baccharin, and Drupanin, exhibited significant protective effects against DSS-induced damage. Notably, the protective effects were consistently observed at relatively low doses, highlighting the high biological activity of these natural compounds. Taken together, these results support the therapeutic potential of Brazilian green propolis-derived molecules as multitarget agents capable of modulating both peripheral and central inflammatory pathways. Although additional mechanistic and translational studies are still required, the present work provides experimental evidence supporting prenylated p-Coumaric acid derivatives as promising candidates for the development of novel therapeutic strategies targeting the gut–brain axis.

## Figures and Tables

**Table 1:** Colonic oxidative stress biomarkers in DSS-induced colitic mice treated with p-Coumaric acid derivatives.

Groups	SOD	CAT	GSH	GST	MDA
Naive	0.2398 ± 0.02	41.60 ± 7.3	39.60 ± 2.24	3.596 ± 0.13	5.821 ± 261.7
Vehicle	0.0810 ± 0.04 ^a^	8.703 ± 1.35 ^b^	28.79 ± 0.42 ^b^	24.51 ± 4.7 ^c^	10.094 ± 516.5 ^d^
ARC 3	0.2933 ± 0.04 ^f^	136.2 ± 19.7 ^c,h^	44.97 ± 3.96 ^f^	1.311 ± 0.65 ^h^	4.359 ± 533.1 ^h^
BAC 3	0.2762 ± 0.03 ^g^	4.939 ± 1.4 ^b^	48.90 ± 2.28 ^a,h^	7.353 ± 0.94 ^g^	2.760 ± 256.8 ^h^
DRU 3	0.3281 ± 0.05 ^g^	34.10 ± 4.8	59.45 ± 4.14 ^c,h^	3.012 ± 0.45 ^h^	2.464 ± 830.1 ^h^

Oxidative stress and inflammatory parameters, including superoxide dismutase (SOD), catalase (CAT), reduced glutathione (GSH), glutathione S-transferase (GST) and malondialdehyde (MDA), were evaluated in colon of non-colitic (Naive), vehicle-treated colitic, and treated groups with Artepillin C (ART-C), Baccharin (BAC), and Drupanin (DRU) (3 mg/kg, p.o.) following dextran sulfate sodium (3% DSS) administration. Data are expressed as mean ± SEM (N = 3–4). Statistical analysis was performed using one-way ANOVA followed by Tukey’s post hoc test. Superscript letters indicate statistically significant differences. Letters a–d represent comparisons versus the Naive (non-colitic) group, and letters f–h represent comparisons versus the vehicle-treated colitic group: a (*p* < 0.05); b, f (*p* < 0.01); c, g (*p* < 0.001); d, h (*p* < 0.0001).

**Table 2:** Cortical inflammatory and oxidative stress biomarkers in DSS-induced colitic mice treated with p-coumaric acid derivatives.

Groups	SOD	CAT	GSH	GST	MDA
Naive	0.2276 ± 0.03	49.23 ± 6.0	45.77 ± 0.67	1.456 ± 0.07	9.029 ± 371.4
Vehicle	0.1445 ± 0.01	6.142 ± 2.57 ^b^	28.14 ± 0.48 ^d^	5.834 ± 1.07 ^b^	13.873 ± 602.6 ^c^
ARC 3	0.2079 ± 0.01	8.803 ± 1.25	38.23 ± 3.66 ^e^	3.265 ± 0.26	9.305 ± 445.7 ^g^
BAC 3	0.1087 ± 0.01 ^a^	513.8 ± 95.7 ^b,g^	35.62 ± 1.9 ^d,g^	3.564 ± 0.27	8.931 ± 641.7 ^g^
DRU 3	2.758 ± 0.15 ^d,h^	138.5 ± 2.56 ^d,h^	37.97 ± 2.7 ^a,f^	4.127 ± 0.72	8.337 ± 830.8 ^h^

Oxidative stress and inflammatory parameters, including superoxide dismutase (SOD), catalase (CAT), reduced glutathione (GSH), glutathione S-transferase (GST) and malondialdehyde (MDA), were evaluated in the cortex of non-colitic (Naive), vehicle-treated colitic, and treated groups with Artepillin C (ART-C), Baccharin (BAC), and Drupanin (DRU) (3 mg/kg, p.o.) following dextran sulfate sodium (3% DSS) administration. Data are expressed as mean ± SEM (N = 3–4). Statistical analysis was performed using one-way ANOVA followed by Tukey’s post hoc test. Superscript letters indicate statistically significant differences. Letters a–d represent comparisons versus the Naive (non-colitic) group, and letters e–h represent comparisons versus the vehicle-treated colitic group: a, e (*p* < 0.05); b, f (*p* < 0.01); c, g (*p* < 0.001); d, h (*p* < 0.0001).

**Table 3:** Hippocampal inflammatory and oxidative stress biomarkers in DSS-induced colitic mice treated with p-coumaric acid derivatives.

Groups	SOD	CAT	GSH	GST	MDA
Naive	0.3109 ± 0.05	81.02 ± 26.5	27.90 ± 0.13	1.698 ± 0.08	6.045 ± 792.9
Vehicle	0.2401 ± 0.08	4.268 ± 1.4	28.24 ± 0.63	3.685 ± 0.78 ^a^	11.724 ± 613.7 ^d^
ARC 3	0.4319 ± 0.07	23.70 ± 1.23	25.81 ± 0.80	3.731 ± 0.51 ^a^	9.305 ± 315.1 ^e^
BAC 3	0.1380 ± 0.01	448.5 ± 52.6 ^d,h^	27.11 ± 0.50	5.759 ± 1.15 ^a^	5.556 ± 100.3 ^f^
DRU 3	3.857 ± 0.51 ^d,h^	33.18 ± 5.73	30.37 ± 2.2	3.202 ± 0.78	4.397 ± 746.8 ^f^

Oxidative stress and inflammatory parameters, including superoxide dismutase (SOD), catalase (CAT), reduced glutathione (GSH), glutathione S-transferase (GST) and malondialdehyde (MDA), were evaluated in the hippocampus of non-colitic (Naive), vehicle-treated colitic, and treated groups with Artepillin C (ART-C), Baccharin (BAC), and Drupanin (DRU) (3 mg/kg, p.o.) following dextran sulfate sodium (3% DSS) administration. Data are expressed as mean ± SEM (N = 3–4). Statistical analysis was performed using one-way ANOVA followed by Tukey’s post hoc test. Superscript letters indicate statistically significant differences. Letters a and d represent comparisons versus the Naive (non-colitic) group, and letters e, f and h represent comparisons versus the vehicle-treated colitic group: a, e (*p* < 0.05); f (*p* < 0.01); d, h (*p* < 0.0001).

## Data Availability

Data generated and analyzed in this study are available from the authors upon request.

## Supplementary Materials

The following supporting information can be downloaded at: https://www.mdpi.com/article/10.3390/ijms27135929/s1.

## Abbreviations

The following abbreviations are used in this manuscript:

ACh	Acetylcholine
AD	Alzheimer’s disease
AHRs	Aryl Hydrocarbon Receptors
ANOVA	Analysis of variance
ART-C	Artepillin C
BAC	Baccharin
BBB	Blood–Brain Barrier
CAT	Catalase
CD68	Cluster of Differentiation 68
CNS	Central nervous system
DAI	Disease activity index
DRU	Drupanin
DSS	Dextran sulfate sodium
EB	Evans Blue Dye
EPM	Elevated Plus Maze
ERK	Signal-Regulated Kinase
FITC-d	Fluorescein Isothiocyanate-Dextran
GI	Gastrointestinal
GSH	Reduced Glutathione
GST	Glutathione S-Transferase
H&E	Hematoxylin and Eosin
HOCl	Hypochlorous Acid
Iba-1	Ionized calcium-binding adapter molecule 1
IBD	Inflammatory Bowel Disease
IFN-γ	Interferon gamma
ILC3s	Innate Lymphoid Cells Type 3
IL-1β	Interleukin-1 Beta
IL-22	Interleukin-22
I.V.	Intravenous
nLNPs	Lipidic Nanoparticles
LPS	Lipopolysaccharide
MDA	Malondialdehyde
MLCK	Myosin Light Chain Kinase
M-MDSCs	Monocyte-Derived Myeloid Suppressor Cells
MPO	Myeloperoxidase
P.O.	Per Oral
RI	Recognition Index
ROS	Reactive Oxygen Species
SEM	Standard Error of the Mean
SOD	Superoxide Dismutase
TJ	Tight Junctions
TNF-α	Tumor Necrosis Factor Alpha
UC	Ulcerative Colitis
