# Supplementary figures and images for: Prenylated p-Coumaric Acid Derivatives Mitigate Neurobehavioral and Neuroinflammatory Alterations Associated with Experimental Colitis
Source: Int J Mol Sci. 2026 Jul 1;27(13):5929. doi: 10.3390/ijms27135929 (PMC13362371; doi:10.3390/ijms27135929)

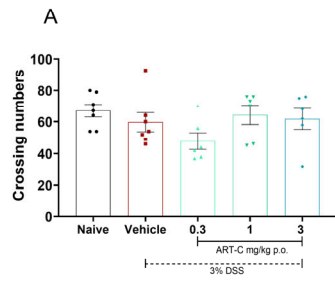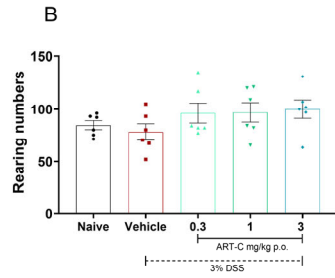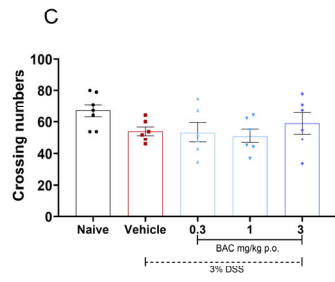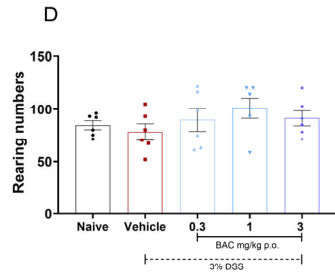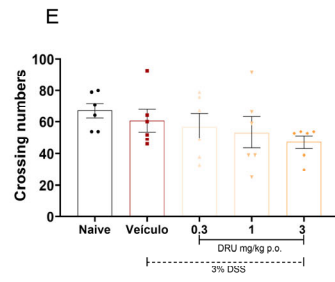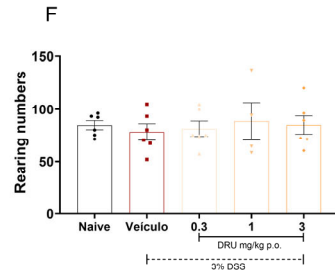

Supplement: Supplementary file 1 [file ijms-27-05929-s001.zip › ijms-4357400-supplementary.pdf]
